# Human Langerhans Cells Control Th Cells via Programmed Death-Ligand 1 in Response to Bacterial Stimuli and Nickel-Induced Contact Allergy

**DOI:** 10.1371/journal.pone.0046776

**Published:** 2012-10-09

**Authors:** Manuel Hitzler, Otto Majdic, Guido Heine, Margitta Worm, Grit Ebert, Andreas Luch, Matthias Peiser

**Affiliations:** 1 German Federal Institute for Risk Assessment (BfR), Department of Product Safety, Berlin, Germany; 2 Institute for Immunology, Medical University, Vienna, Austria; 3 Department of Dermatology and Allergy, Allergy-Center-Charité, CCM, Charité-University Medicine Berlin, Berlin, Germany; 4 Department of Human Molecular Genetics, Max Planck Institute for Molecular Genetics, Berlin, Germany; Wayne State University, United States of America

## Abstract

Langerhans cells (LCs) are suspected to initiate inflammatory immune responses to contact allergens and pathogenic bacteria. In chronic infectious diseases, programmed death ligand (PD-L) 1 exhibits both inhibitory and costimulatory functions on T cell-mediated activation and tolerance. Here, we investigated the effects of contact allergens and bacterial stimuli on PD-L1 expression in LCs and the effects of altered PD-L1 expression on cytokine release of subsequently cocultured T cells. Monocyte-derived LCs (MoLCs), LCs, and skin sections of patients suffering from allergic contact dermatitis were challenged with nickel and then analyzed for PD-L1 expression by confocal laser scanning microscopy and flow cytometry. In blocking experiments, we found that the release of Th cell specific cytokines was dependent on both stimulation of LCs and inhibition of PD-L1-PD-1 interactions. Stimulation with peptidoglycan (PGN) or lipopolysaccharide (LPS) and blockage of PD-L1 with a specific antibody triggered the release of high levels of IL-17, IL-22, TNF-α, and IFN-γ in CD4^+^T cells. If nickel was used as a stimulus, blockage of PD-L1 led to high amounts of TNF-α and IL-22. A closer look revealed PD-L1-dependent upregulation of IL-17 secretion in FACS-sorted CCR6^+^/CCR4^+^ T memory cells. In the presence of anti-PD-L1, PGN induced secretion of IFN-γ and IL-17 in total CCR6^+^ cells, while nickel triggered secretion of IFN-γ and IL-17 exclusively in CCR6^+^/CCR4^+^ cells. Our findings suggest that PD-L1 on LCs plays a crucial role in type IV allergic reactions and in response to bacterial stimuli by controlling the nature of inflammatory Th cell responses.

## Introduction

Inflammatory responses to bacterial pathogens and allergic contact dermatitis (ACD) have in common that distinct subsets of Th cells are crucially involved. Whereas the involvement of cytotoxic T cells becomes clearly evident in light of the skin lesions induced, nearly all functional subsets of CD4^+^Th cells could also be involved in allergic reactions. In addition to Th1 and Th2 cells [1]–[3], in the human system Th17 and Th22 were reported to express specific cytokine/chemokine receptors and transcription factors [4], [5]. IL-22 and IL-17 have been shown to mediate protection and defense against bacteria such as *Citrobacter rodentium* and *Klebsiella pneumoniae*, and *Mycobacterium tuberculosis* and *Bordetella pertussis*, respectively [6]–[9]. In experimental models even parts of the bacterial cell wall, such as LPS, were shown to induce IL-22 secretion in T cells [4]. Little is known on the exact role of these Th cell subpopulations in inflammatory skin diseases.

It has been suggested that ACD can be distinguished from other skin disorders like psoriasis and atopic eczema by the specific profile of Th cell subsets involved and Th17 and Th22 were detected in skin lesions of allergic individuals [10], [11]. Among environmental contact allergens, nickel is the most important. A new aspect in the mechanism of allergic diseases has been disclosed by the finding that nickel and even the respiratory allergen Der p 2 provide proinflammatory stimuli through facilitating TLR4 signaling [12], [13]. Recently, polarization of Th17 in response to LPS-containing allergens has been observed in a murine model [14]. In addition to other costimulatory molecules such as CD86, programmed death ligand (PD-L)1 (B7-H1, CD274) and PD-L2 bind to their cognate receptor PD-1 on activated T and B cells, thereby modulating and fine tuning the balance of immune responses toward pathogens and autoimmune tissue damage [15], [16]. PD-L1 controls the T cell stimulatory potential by limiting IL-2 and IFN-γ release [17], [18]. There is strong evidence for further direct effects of PD signaling on T cell responses such as proliferation, cytolytic activity and exhaustion [19].

In this study we explored PD-L1 expression in human skin biopsies from allergic individuals after challenging with nickel. Our data on LC-Th cell interactions suggest that PD-L1 expressed on the surface of LCs plays a regulatory role in skin`s immune response to bacteria and in ACD by limiting the release of proinflammatory cytokines from Th cells.

## Materials and Methods

### Ethical Approval

For skin biopsies, obtained from patients with a history of nickel mediated ACD, and for skin samples received from plastic surgery we obtained approval by the ethics committee of the Charité - Universitätsmedizin Berlin (ethics-approval no. SI.246). All participants provided their written informed consent to donate their skin for research purposes. Anonymized blood samples were obtained from the German Red Cross blood donation service Berlin with informed written consent from all participants. All studies have been approved by the Institutional Review Board (SFP-Committee of BfR) and were in accordance to the Helsinki guidelines. No part of these studies was conducted outside of Germany.

**Figure 1:**
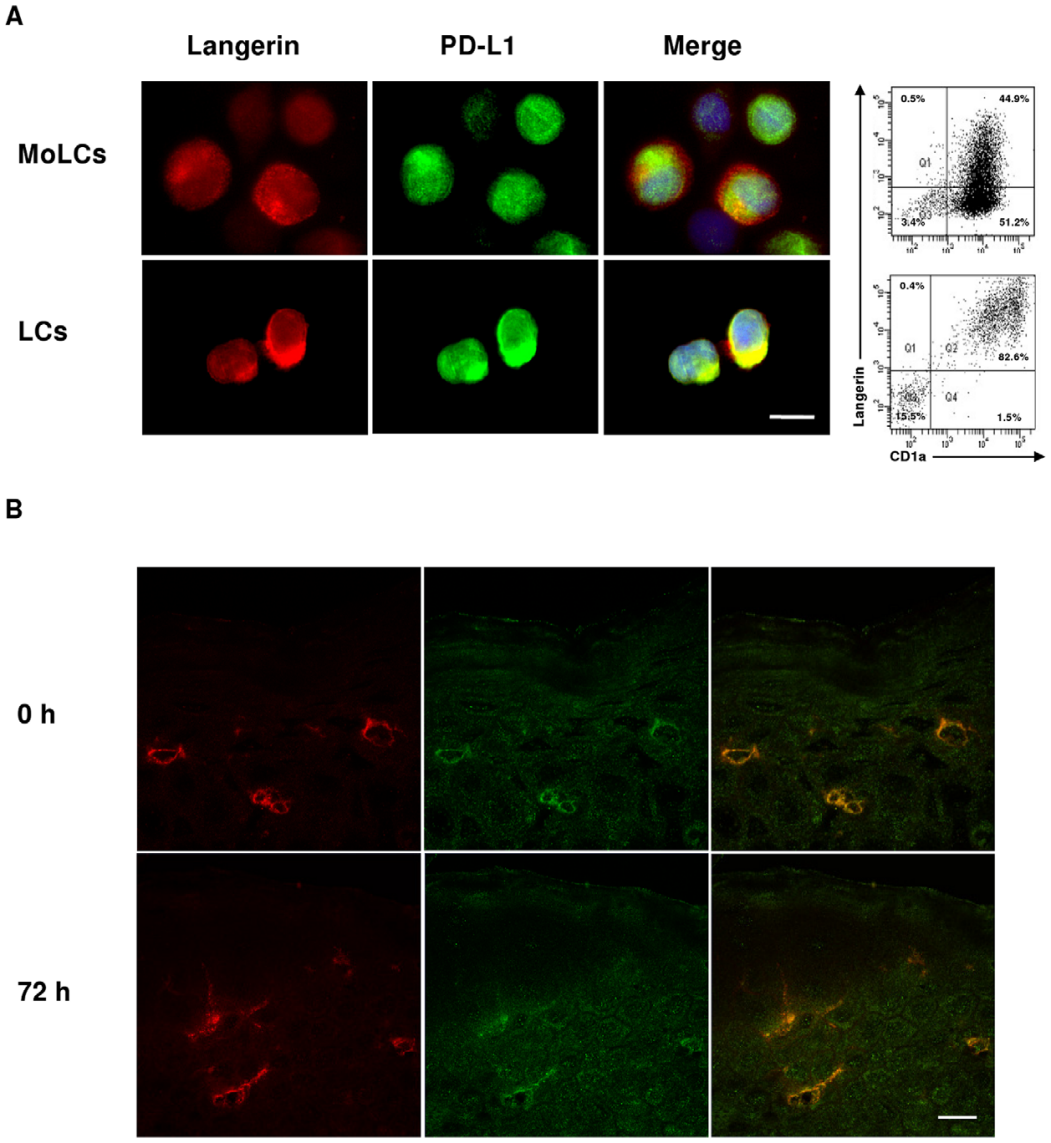
PD-L1 is expressed on isolated LCs and in LCs *in situ* of ACD patients. A. Left side: Immunofluorescence microscopy analysis of the expression of langerin and PD-L1 on the surface of human MoLCs and LCs derived from blood monocytes and epidermal tissue of healthy donors, respectively. Right side: Expression of langerin and CD1a in MoLCs (upper chart) and LCs (lower chart) detected by flow cytometry. Bar = 10 µm. B. Expression of HLA-DR and PD-L1 in skin sections of ACD donors before and 72 hours after challenge with 5% NiSO_4_, as detected by confocal laser scanning microscopy. Representative images of two stack series in the epidermal layer are shown. Bar = 10 µm.

### Cell Isolation and Culture

Mononuclear cells were prepared from buffy coats of samples from healthy adult donors by Ficoll gradient (PAA Laboratories, Pasching, Austria). Monocytes were isolated by plastic adherence and differentiated in RPMI 1640 supplemented with 2 mM L-glutamine, 100 U/ml penicillin, 100 mg/ml streptomycin, and 10% v/v heat-inactivated FBS (PAN Biotech, Aidenbach, Germany). Cells were kept in a 6-day culture in the presence of human recombinant GM-CSF (100 ng/ml), IL-4 (10 ng/ml, ImmunoTools, Friesoythe, Germany), and TGF-β1 (10 ng/ml, R&D Systems, Wiesbaden-Nordenstadt, Germany) according to a previous protocol [20]. CD4^+^T cells were isolated from density gradient enriched PBMCs by negative selection using CD4^+^T cell isolation Kit II. For analyses of T memory cells CD4^+^CD45^+^T cells were isolated from PBMCs by negative selection using memory CD4^+^T cell isolation Kit (all Miltenyi, Bergisch-Gladbach, Germany). Cells were stained for 30 minutes with PE-Cy7-labeled anti-CCR4 (1G1) and PE-labeled anti-CCR6 (11A9; all BD Biosciences, Heidelberg, Germany). T memory subpopulations were isolated by an Aria III cell sorter (BD Biosciences, Heidelberg, Germany). Normal human skin was obtained from surgical breast reductions and single cell suspensions were prepared [21]. Briefly, epidermal sheets were peeled off the dermis after dispase treatment (2 U/ml, Roche, Mannheim, Germany). Epidermal disintegration was conducted by trypsin (0.25%; Biochrom, Berlin, Germany) in the presence of DNase I (20 U/ml, Boehringer, Germany). LCs were isolated from total epidermal cells by MACS using anti-CD1c (AD5-8E7, IgG2a) and anti-biotin coated microbeads (Bio3-18E7, IgG1, Miltenyi).

**Figure 2:**
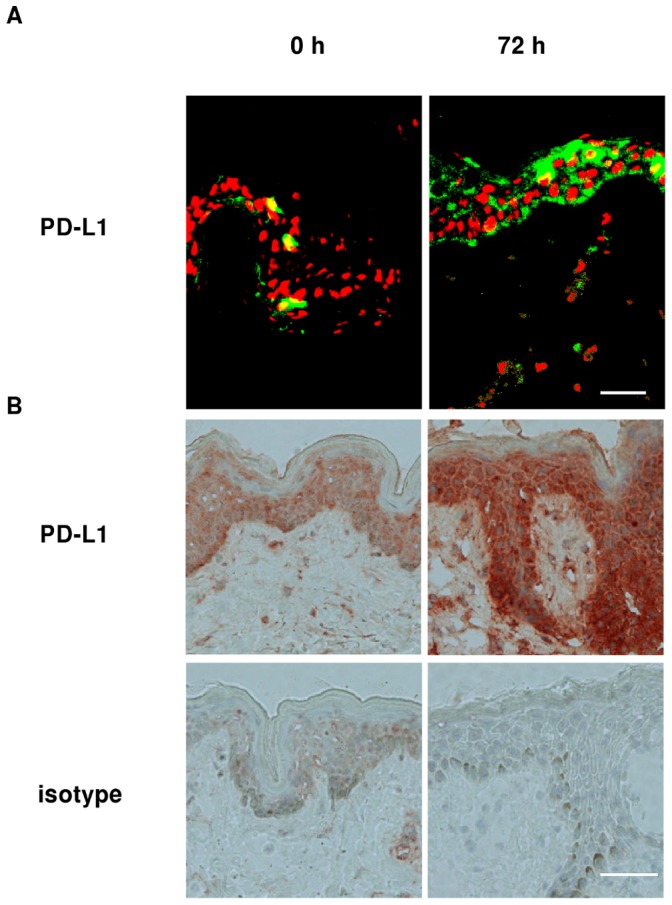
Intraepidermal PD-L1 increases in ACD patients after challenge with nickel. A. PD-L1 and nuclei are visualized by Alexa Fluor 488 (green) and 7-AAD (red) immunofluorescence in sections from lesions of ACD patients after being exposed to 5% NiSO_4_, for 0 hours and 72 hours. Bar = 50 µm. B. Biopsies taken from inflamed skin tissue areas of ACD donors. The sections were incubated with anti-PD-L1, stained with EnVision+ System-HRP AEC, and counterstained with hematoxylin. Representative light microscopy images covering the profile from the upper *stratum corneum* down to dermal rete pags are depicted. Bar = 50 µm.

### Blockade Experiments in T Cell Assays

10^4^ MoLCs were treated with 20 µg/ml PGN (InVivogen, San Diego, CAL), 1 µg/ml LPS (Fluka, St. Louis, MO) or 200 µM nickel(II)sulfate (NiSO_4_) (Sigma Aldrich, St. Louis, MO) for 24 hours. Then 10^5^ allogeneic CD4^+^T cells were added and cells were cocultured in 200 µl Xvivo15 (Lonza, Verviers, Belgium) in U-bottom plates for additional 6 days. For PD-L1 and CD86 blockage either of the following antibodies or fusion proteins was added to MoLCs 30 minutes before being pooled with CD4^+^T cells: 10 µg/ml of anti-PD-L1 (clone 5-272; generated in the laboratory of O.M.), anti-CD86 (clone IT2.2), IgG1 isotype (BD Biosciences, Heidelberg, Germany), 10 µg/ml of B7-H1/PD-L1 Fc chimera, or IgG1 Fc (R&D Systems). On day-7 supernatants were collected and analyzed for IL-17, IL-22, IFN-γ and TNF-α by ELISA (DuoSet, R&D Systems).

### Activation of MoLCs

10^5^ cells were incubated with NiSO_4_, 2,4-dinitrochlorobenzene (DNCB; Sigma) or sodium dodecyl sulphate (SDS; ROTH, Karlsruhe, Germany; individual concentrations as indicated), 1 µg/ml LPS or 20 µg/ml PGN in RPMI 1640. Expression of PD-L1, HLA-DR and CD86 was analyzed by FACS 48 hours later. Preparations of NiSO_4_ and DNCB contained endotoxin below 0.1 EU/µg compound, as determined by using the Limulus amebocyte lysate assay (BioWhittaker, Walkersville, MD, USA).

**Figure 3:**
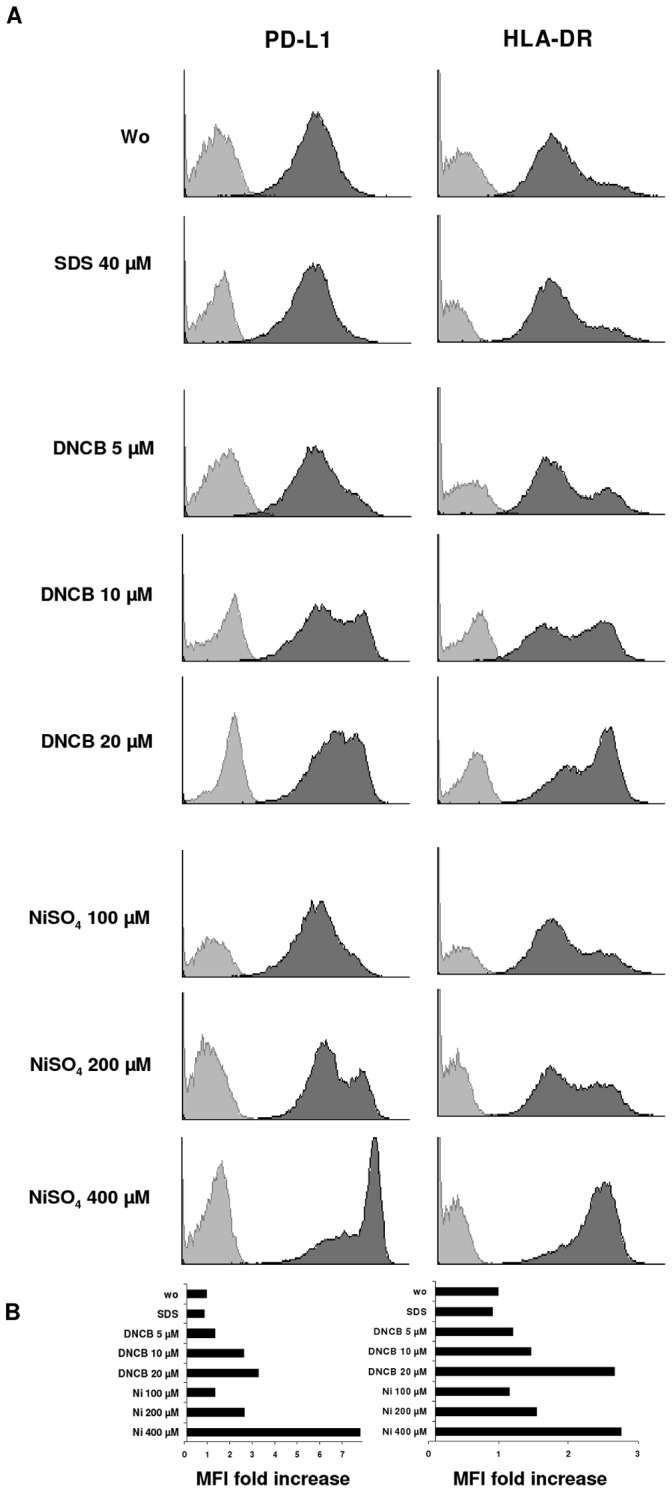
PD-L1 and HLA-DR are upregulated in MoLCs after stimulation with allergens. MoLCs were exposed to graded doses of DNCB, nickel or SDS for 24 hours and stained by anti-PD-L1 and anti-HLA-DR, respectively. FACS analyses were gated on viable cells. A. Protein expression is shown as dark gray graphs in overlay histograms. Cells stimulated under the same conditions were stained with isotype-controls and are represented by light gray. B. Bars indicate fold increase of mean fluorescence intensities (MFI) compared to untreated control. Results shown are representative for four independent experiments with different donors.

### Flow Cytometry

Surface markers were stained with PE-labeled anti-langerin (DCGM4, Immunotech, Marseille, France), APC-labeled anti-CD1a (HI149), FITC-labeled anti-CD86 (FUN-1), PE-labeled anti-PD-L1 (MIH1), and APC-H7-labeled anti-HLA-DR (L243, all BD Biosciences, Heidelberg, Germany) or with isotype controls. Cells were analyzed in the FACS Canto II (BD Biosciences, Heidelberg, Germany) flow cytometer using FACSDiva software.

### Fluorescence Microscopy, Immunohistochemistry, Laser Scanning Microscopy

Cells were fixed and permeabilized in 3.7% paraformaldehyde and 0.2% Triton-X for 30 minutes on poly-L-lysine (Sigma-Aldrich, St. Louis, MO, USA) coated coverslips. After blocking with 1% BSA in PBS cells were incubated with primary antibody for 1 hour followed by goat anti-mouse Alexa Fluor 488 antibody. Nuclei were counterstained with 7-AAD (all Invitrogen, Darmstadt, Germany). Skin samples were obtained from patients with a history of nickel-mediated ACD. All participants gave informed consent, and the study was approved by the local medical ethics committee. Nickel challenge was performed with 5% NiSO_4_ in vaseline according to international guidelines in 4 mm finn chambers (Almirall, Reinbek, Germany). Biopsies were taken before or 1 to 3 days after nickel exposure and immediately snap frozen. The samples were blocked with 1% BSA in PBS. After incubation with mouse anti-human PD-L1 samples were stained with EnVision+ System-HRP AEC (Dako, Hamburg, Germany) according to manufacturer’s protocol. In brief, tissue sections were incubated with labeled polymer-HRP anti-mouse for 30 minutes followed by 30 minutes of incubation with AEC+ chromogen. Sections were counterstained with Mayer’s Hematoxylin Lillie’s Modification (Dako) and analyzed by light microscopy. For confocal laser scanning microscopy sections were incubated for 1 hour with mouse anti-human PD-L1, rat anti-human langerin (Dendritics, Lyon, France) and rat anti-human HLA-DR (Abcam, Cambridge, UK), and stained with goat anti-mouse Alexa Fluor 488 and goat anti-rat Alexa Fluor 546 conjugated antibodies (Invitrogen) for 1 hour. The thickness of all skin sections was 6 µm.

### Statistical Methods

Mean value, standard deviation (SD) and statistical significance were calculated using SigmaPlot (Systat, Erkrath, Germany). For analysis of differences between experimental groups, two tailed Student’s t test for paired data was used. Values of p≤0.05 were considered statistically significant.

**Figure 4:**
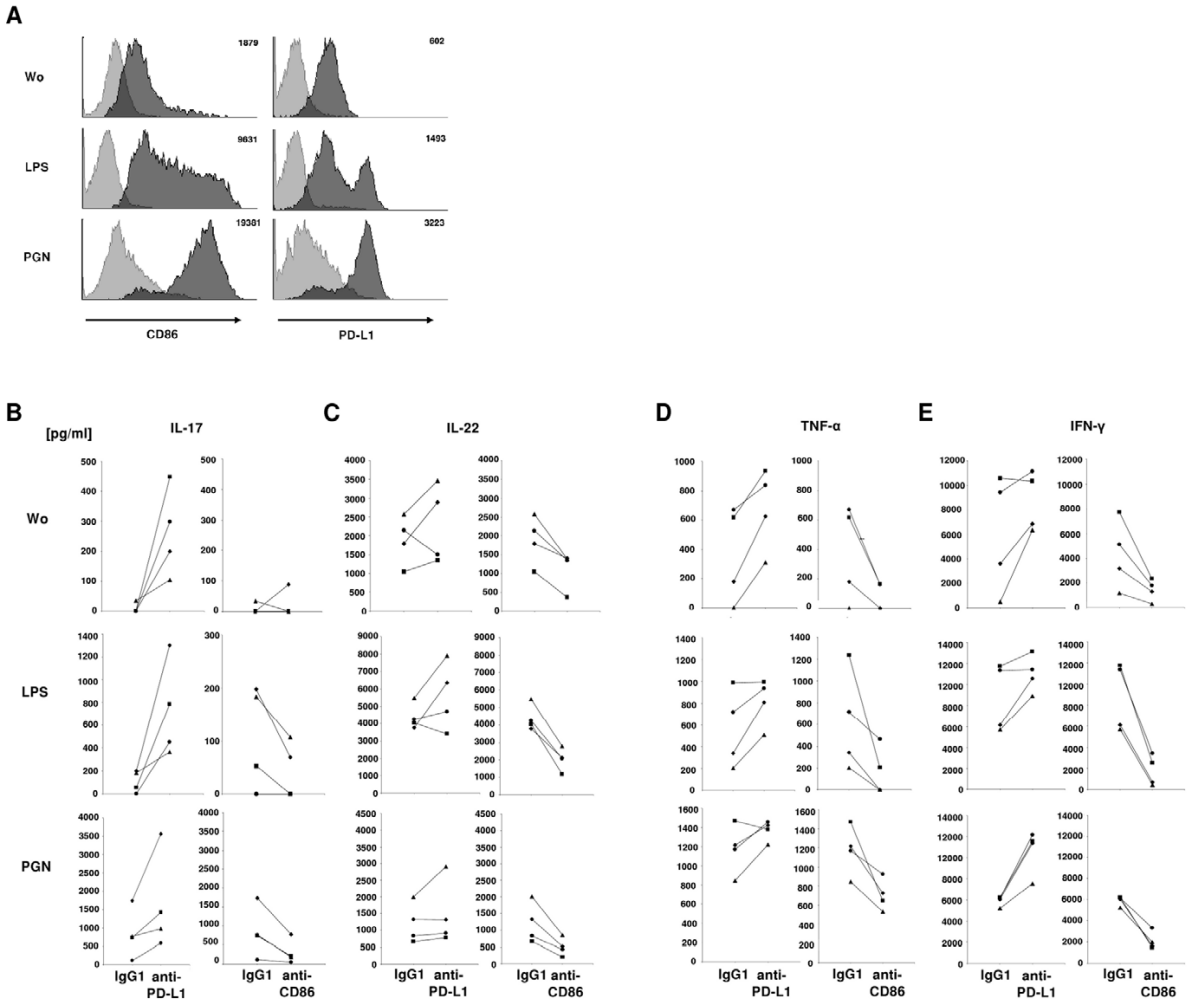
Blockage of PD-L1 enhances cytokine release from stimulated Th cells. A. FACS analyses of CD86 or PD-L1 on MoLCs, dark gray, or isotype-controls, light gray. Numbers depicted in the histograms show mean fluorescence intensities. MoLCs were stimulated by LPS or PGN for 24 hours. Histograms shown are from the same donor representative for results obtained with three different donors. B **–** E. Secretion of cytokines by CD4^+^T cells in coculture with MoLCs. MoLCs were stimulated by LPS or PGN (as in A.), and incubated with IgG1, anti-PD-L1, or anti-CD86. Secretion of IL-17, IL-22, TNF-α, and IFN-γ in pg/ml was detected via ELISA 7 days after starting the coculture. Data shown are from 4 different donors, indicated by the following symbols: ▴, ▪, •, ♦. Wo, without stimulus.

## Results

### PD-L1 is Expressed in ACD Biopsies and Colocalizes with Langerin and HLA-DR

Initial *in vitro* screening for protein markers revealed increased expression levels of PD-L1 in MoLCs upon treatment with TLR agonists and also after exposure to nickel. To evaluate the role of PD-L1 in chemically induced ACD, PD-L1 and langerin expression was analyzed in skin tissue. Since the viability of epidermal LCs rapidly decreases upon tissue digestion and transfer into cell culture, we also generated LC-like cells from PBMCs as reported [20]. In LCs and MoLCs PD-L1 was found to colocalize with langerin, with bright signals for PD-L1 especially in epidermal LCs isolated from healthy individuals (Figure 1A). Furthermore, in skin biopsy sections derived from local lesions of ACD patients constitutive PD-L1 expression was demonstrated in HLA-DR positive cells located in the suprabasal and upper epidermal layers (Figure 1B). In these sections the particular keratinocyte interstices highlighted by anti-HLA-DR turned out to be filopodia of LCs embedded between neighboring keratinocytes. Compared to unchallenged skin from ACD patients treatment with nickel for 72 hours led to a further increase in sizes of cell bodies and dendritic protrusions of HLA-DR positive LCs.

**Figure 5:**
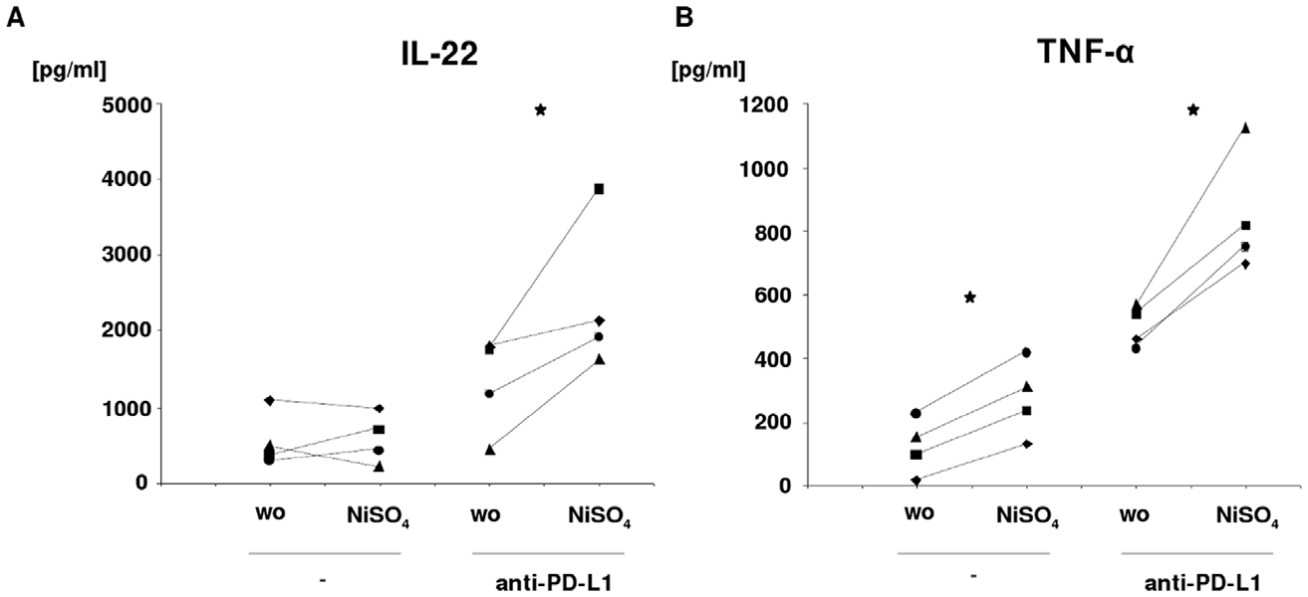
Nickel induces release of IL-22 and TNF-α after blockage of PD-L1. A., B. Secretion of cytokines by CD4^+^T cells while being in coculture with MoLCs. MoLCs were either stimulated by nickel for 24 hours (+) or not (−), and incubated with anti-PD-L1 (+) or not (−). Protein secretion was detected via ELISA in the supernatants 7 days after starting the coculture of MoLCs with allogeneic CD4^+^T cells. Secretion levels (in pg/ml) are shown for IL-22 and TNF-α. Data shown are from 4 different donors and visualized by scatter plots; donors 1–4 are indicated by the following symbols: ▴, ▪, •, ♦.

### Epidermal PD-L1 Increases after Challenge with Nickel

PD-L1 tissue expression was analyzed by immunohistochemistry in tissue biopsies taken from ACD patients after nickel exposure (Figure 2). By fluorescence microscopy PD-L1 expression was found greatly enhanced upon challenge with nickel. At the 72 hours time point the majority of cells constituting epidermal layers exhibited strong expression of PD-L1. By contrast, comparably low PD-L1 levels were observed in biopsies of unchallenged ACD patients (time point: 0 h).

### Nickel and DNCB Elevate PD-L1

We next questioned whether PD-L1 has a regulatory function in fine tuning ACD effector cell responses. Langerin^+^MoLCs were exposed to graded doses of 2,4-dinitrochlorobenzene (DNCB), nickel or to the irritant sodium dodecyl sulphate (SDS) as control (Figure 3). Both DNCB and NiSO_4_ dose-dependently led to increases in PD-L1 levels, with highest expression at 400 µM NiSO_4_ and at unaffected viability. HLA-DR was also found dose-dependently upregulated (Figure 3). In comparison to HLA-DR, upregulation of PD-L1 in response to chemical allergens was slightly more pronounced. The irritant (SDS) control confirmed the specificity of this response.

**Figure 6:**
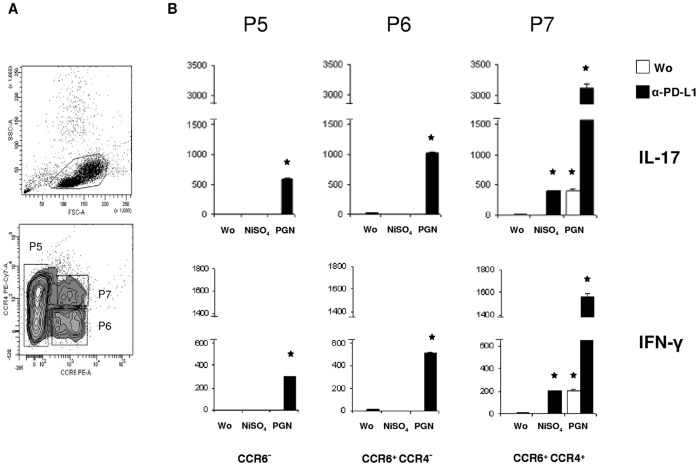
PGN and nickel induce IFN-γ and IL-17 in CCR6^+^ and CCR6^+^/CCR4^+^ cells. T memory cells were incubated with MoLCs pretreated with anti-PD-L1 (+) or not (−). A. Expression of CCR6 and CCR4 in CD4^+^T cells enriched from the blood of healthy donors by depletion of non-CD4 and naïve (CD45RA^+^)T cells. T memory subpopulations were gated as CCR6^–^ (P5), CCR6^+^/CCR4^–^ (P6), or CCR6^+^/CCR4^+^ (P7) cells. B. Release of IFN-γ and IL-17 by sorted subpopulations. MoLCs were stimulated by nickel or PGN for 24 hours and incubated or not with anti-PD-L1. Secretion levels (in pg/ml) are shown for IFN-γ and IL-17 7 days after starting the coculture. Data reported as mean +/− SD of triplicates are representative for 3 different donors. Wo, without stimulus.

### Blockade of PD-L1 on Bacterial Stimulated MoLCs Induces Th Cell Cytokines

The influence on subsequent effector T cell responses was addressed by PD-L1 neutralization studies. In addition to TCR signals and cytokines, costimulatory molecules could induce a specific pattern of Th cells that contribute to ACD or defense against pathogens. Blocking studies in mice showed that these surface markers essentially contribute to hapten-induced contact sensitivity reactions [22]–[24]. Recently it could be shown that TLR agonists or TLR-associated proteins may be mimicked by allergens [13], [25]. Thus we examined the effect of TLR2 and TLR4 agonists PGN and LPS on Th cell polarization. Strong upregulation of CD86 and PD-L1 on MoLCs could be demonstrated for PGN and LPS (Figure 4A). To evaluate the impact of PD-L1 on responses of Th cells while being in coculture with MoLCs, PD-L1 and CD86 were blocked by specific antibodies. MoLCs stimulated by LPS failed to induce significant amounts of IL-17 during coculture with allogeneic CD4^+^T cells (Figure 4B). By contrast, the amounts of IL-17 reached high levels when LPS- or PGN-activated MoLCs were first treated with anti-PD-L1. In contrast, blockage of CD86 on the surface of LPS- or PGN-stimulated MoLCs led to a significant decrease of the amounts of IL-17 in MoLC-Th cell cocultures when compared to non-blocked CD86-expressing MoLCs (Figure 4B). These data led us to hypothesize that PD-L1 would be in general an important regulator in the control of proinflammatory cytokines secreted by specific Th cell subpopulations. Hence we analyzed the secretion of IL-22, TNF-α and IFN-γ, each of which is known to contribute to certain forms of skin pathogenesis. In contrast to IL-17, basal secretion levels of these cytokines were already high, but could still be increased if MoLCs were pretreated with LPS or PGN prior to pooling with T cells (Figure 4C–E). Remarkably, antibody-mediated blockage of PD-L1 led to strong increases of these cytokines in the supernatants of MoLC-Th cell cocultures. Further experiments using PD-L1 Fc protein that targets the PD-1 receptor on the surface of T cells confirmed the data obtained in experiments using anti-PD-L1. PD-L1 Fc chimera led to strong and significant increases of the levels of IL-22, TNF-α, and IFN-γ in the supernatants of subsequent MoLC-Th cell cocultures (Figure S1).

### PD-L1 Controls IL-22 and TNF-α in Nickel-activated CD4^+^T Cells

If TLR agonists trigger the expression of PD-L1 on the surface of LCs, and PD-L1 becomes crucial in controlling (i.e. restricting) Th cell polarization, then the strong contact allergen nickel, as potent inducer of PD-L1 expression, could be expected to exert similar effects. Consequently, the influence of nickel on the release of Th cell-derived cytokines in MoLC-T cell cocultures was addressed. MoLCs were pre-activated by nickel and, after incubation with anti-PD-L1, cocultured with CD4^+^T cells. Only for basal secretion levels of TNF-α, single administration of nickel induced an increase in protein levels (Figure 5B). For IL-22 (Figure 5A) and IL-17 and IFN-γ (not shown), basal secretion levels were low and stayed low even after stimulation of MoLCs by nickel (Figure 5A). However, antibody-mediated blockage of PD-L1 prior to coculture significantly increased the levels of TNF-α and IL-22 when compared to non-blocked or non-stimulated conditions. This effect was also evident for IL-17 and IFN-γ, but unreached the statistical significance threshold of p≤0.05.

### PD-L1 Restricts IL-17 and IFN-γ in Activated CCR6^+^T Cell Subsets

To examine whether IL-17 and IFN-γ were regulated by PD-L1 in specific Th subpopulations, we further separated total CD4^+^T cells based on their expression of chemokine receptors specific for skin homing T memory cells. Human T cells that have been differentiated *in vitro* toward a distinct memory phenotype were shown to release IL-17 and IFN-γ after unphysiological stimulation [4], [26]. We highly enriched this small T cell subset from pre-sorted CD4^+^T cells by applying combined gating on CCR6 and CCR4 during FACS (Figure 6A). Upon stimulation of MoLCs with anti-PD-L1 and nickel, cocultured CCR6^–^ or CCR6^+^/CCR4^–^ cells did not release considerable amounts of either IL-17 or IFN-γ (Figure 6B). By contrast, a significant release of these cytokines was detectable after co-administration of anti-PD-L1 and PGN in CCR6^+^/CCR4^–^ cells. In particular, extremely high amounts of IL-17 were induced by nickel and anti-PD-L1 in the subset of CCR6^+^/CCR4^+^ cells. In this particular population, however, no elevated secretion of IL-10 and IL-21 was detectable under similar conditions (not shown). Together these data are in favour of the suggested regulatory role of PD-L1 on the polarization and subsequent cytokine release of Th cells after engaging into physical contacts with bacterial- or nickel-activated LCs that express high levels of this surface molecule.

## Discussion

In this study we uncovered a regulatory role of the LC surface marker PD-L1 in ACD and bacterial defense. Recently, PD-L1 was detected on mRNA level in dermal DCs and migratory LCs [27]. We demonstrate protein expression of PD-L1 in human ACD skin biopsies, epidermal LCs and MoLCs in response to contact allergens and its specific inhibitory effects on the secretion of IL-22 and TNF-α by Th cells. These findings further support observations on anti-PD-L1 treated DCs that could elevate IFN-γ in allogenic CD4^+^ T cells [28]. However, this earlier study was not performed in connection with allergen or bacterial stimulation. *In vivo*, contact allergens such as DNCB, 2,4,5-trinitrochlorobenzene (TNCB), oxazolone, and cinnamic aldehyde were reported to modulate the phenotype and function of murine LCs [29]. Apart from direct effects on LCs, the influence on cocultured T cells appeared as enhanced proliferation. Considering this pathbreaking observation made by Aiba and Katz back in 1990, we questioned whether PD-L1 may exhibit a costimulatory function in T cell-mediated bacterial defense and delayed-type hypersensitivity reactions. In skin biopsies of ACD patients we detected high expression of PD-L1 after nickel challenge. Upregulation of PD-L1 in emigrating LCs has been recently reported [27]. We could demonstrate that PD-L1 colocalizes with langerin, thus identifying LCs as PD-L1-expressing cells. DNCB and nickel, but not the irritant SDS, increased the expression levels of PD-L1 and HLA-DR in MoLCs (Figure 3). Since DNCB and nickel were previously proven to augment HLA-DR expression in CD1a^+^MoDCs stronger than any other chemical allergen [30], we assumed these and bacterial compounds being capable of inducing costimulatory signals on LCs that might be transferable to T cells. Applying cocultures we observed significant secretion of IFN-γ, IL-17, IL-22 and TNF-α by CD4^+^T cells if PD-L1 was blocked on LPS or PGN stimulated MoLCs. Previous studies could demonstrate proliferation and IL-22 secretion, but no detection of IL-17 in CD4^+^T cells while being in coculture with LPS matured DCs or CpG matured plasmacytoid DCs [4]. In our experiments, for secretion of IL-17 in response to LPS and PGN blocking of PD-L1 was mandatory. While exposure to complete bacterial cells was shown to suffice for induction of Th17 [8], [9], in experimental models using cell wall compounds of bacteria additional signals besides LPS or PGN were required for Th17 differentiation [31]. Previous studies demonstrated that inhibition of PD-L1, but not PD-L2, enhanced hapten-induced ear swelling. In the presence of anti-PD-L1 (clone MIH5), both 2,4-dinitrofluorobenzene (DNFB)-pulsed antigen-presenting cells and the PD-L1-transfected LC cell line XS106 promoted proliferation of lymph node cells or T cells from naïve mice [32], [33]. In addition, PD-L1^+^XS106 cells suppressed the release of IFN-γ and IL-2 in CD4^+^ and CD8^+^ T cells. These observations support the notion that PD-L1 expression not only becomes enhanced during maturation of LCs at inflammatory conditions, but also controls cytokine production in subsequent adaptive immune responses. It becomes tempting to speculate that the detection of IFN-γ/TNF-α, IL-17 and IL-22 indicates the presence of Th1, Th17 and Th22 cells. Recently it was shown that Th17 and Th22 cells participate in common skin diseases such as ACD, psoriasis and atopic dermatitis [10], [34]. Based on our findings of increased expression levels in epidermal cells of patients suffering from ACD and the restriction of the secretion of TNF-α and IL-22, an important role of PD-L1 in confining an excessive release of cytokines during skin pathogenesis seems obvious and biologically consistent. In addition, PD-L1-mediated regulation on IL-17 is supposed to represent later events in memory T cell development. Furthermore, cytokine regulation may be ensured through a PD-L1-PD-1 signaling feedback loop or a mechanism induced with time delay to endure positive B7 costimulatory signals. Since 2005 several studies have challenged the notion that skin residential LCs play a crucial role in the initiation of adaptive immune responses subsequent to antigen uptake and migration into draining lymph nodes [35]. In mice lacking LCs, surprisingly intensified or diminished but no abrogated contact hypersensitivity reactions were reported [36], [37]. Therefore, LCs might be dispensable for induction of contact hypersensitivity reactions. In contrast to dermal DCs, allergen-challenged LCs reach auricular lymph nodes with delay and then populate in T cell areas rather than in the outer paracortex of B cell follicles [38]. It seems possible that specific localization of LCs in T cell-rich zones of lymph nodes may reflect more likely a function in specific control of cytotoxic T cells and Th cells rather than in initiation of plasma cell differentiation. Congruently, skin reactions upon exposure to contact allergens such as DNFB, TNCB or oxazalone are reported to include cytotoxic T cell and Th cell responses [39]. Along with our results on the effects of PD-L1 in Th cell differentiation and cytokine secretion *in vitro*, the current knowledge indicates that LCs may execute a controlling function in contact allergy, most likely through expression of immunoregulatory members of the B7 protein family including PD-L1. Experiments where LCs were eliminated from murine skin by corticosteroids or tape stripping point to similar conclusions [40]. Instead of LCs, other skin residential DCs may act as relevant antigen-presenting cells in the sensitization phase of contact hypersensitivity reactions. In contrast, LCs more likely induce confining and down-regulatory signaling thereby restricting immune responses to a certain level until a disease-specific threshold of integrated proinflammatory signals has been passed.

Collectively, in this study we present data that suggest a crucial role of PD-L1 in skin’s immune response to bacterial stimuli and in ACD. LPS, PGN, and the ubiquitous contact allergen nickel were shown to be capable of inducing PD-L1 expression on the surface of LCs which in turn limits the release of proinflammatory IL-22 and TNF-α from Th cells. Thus our data may set the course toward a promising approach aimed at inducing PD-L1 during elicitation of ACD by topical administration of specific stimuli like certain TLR agonists. In addition, synthetic analogues could be used to fine-tune PD-L1 expression for improved treatment of inflammatory skin disorders and infectious diseases.

## Supporting Information

Figure S1 **Fusion protein Fc-PD-L1 elevates release of IL-22, TNF-α, and IFN-γ.** CD4^+^T cells were incubated with B7-H1/PD-L1 Fc chimera for 2 hours and subsequently cocultured with allogeneic MoLCs. Protein secretion was detected via ELISA in the supernatants 7 days after starting the coculture of MoLCs with CD4^+^T cells. Data shown are from 3 different donors; donors 1–3 are indicated by the following symbols: ▴, ▪, ♦. Wo, without stimulus.
(TIF)
