# Supplementary figures and images for: Human Langerhans Cells Control Th Cells via Programmed Death-Ligand 1 in Response to Bacterial Stimuli and Nickel-Induced Contact Allergy
Source: PLoS One. 2012 Oct 9;7(10):e46776. doi: 10.1371/journal.pone.0046776 (PMC3467287; doi:10.1371/journal.pone.0046776)

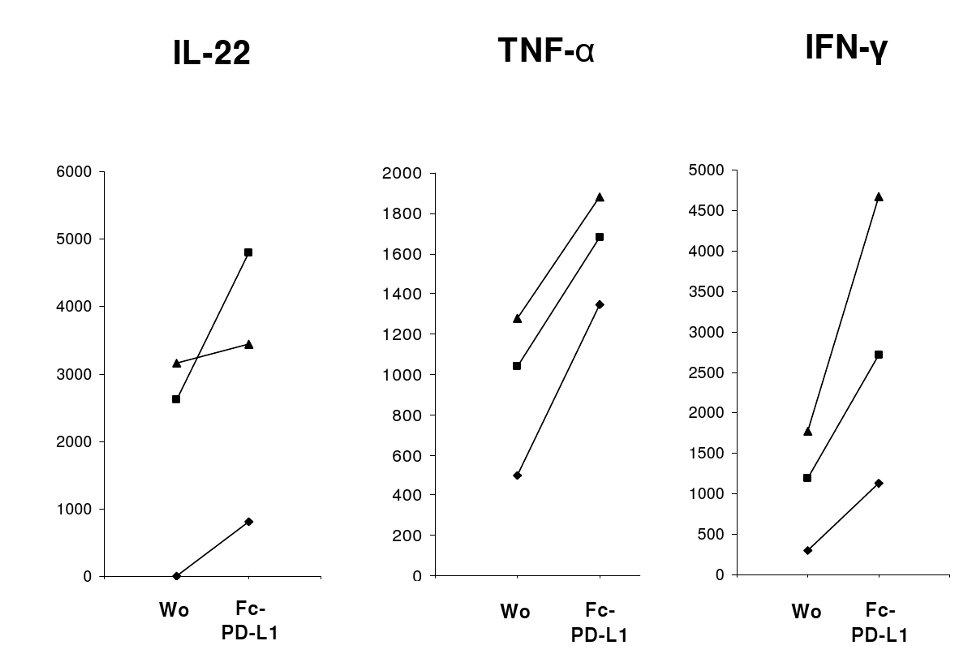

Supplement: Figure S1 — Fusion protein Fc-PD-L1 elevates release of IL-22, TNF-α, and IFN-γ. CD4+T cells were incubated with B7-H1/PD-L1 Fc chimera for 2 hours and subsequently cocultured with allogeneic MoLCs. Protein secretion was detected via ELISA in the supernatants 7 days after starting the coculture of MoLCs with CD4+T cells. Data shown are from 3 different donors; donors 1–3 are indicated by the following symbols: ▴, ▪, ♦. Wo, without stimulus. (TIF) [file pone.0046776.s001.tif]
